# HACER: an atlas of human active enhancers to interpret regulatory variants

**DOI:** 10.1093/nar/gky864

**Published:** 2018-09-24

**Authors:** Jing Wang, Xizhen Dai, Lynne D Berry, Joy D Cogan, Qi Liu, Yu Shyr

**Affiliations:** 1 Center for Quantitative Sciences, Vanderbilt University Medical Center, Nashville, TN, USA; 2 Department of Biostatistics, Vanderbilt University Medical Center, Nashville, TN, USA; 3 Department of Pediatrics, Vanderbilt University School of Medicine, Nashville, TN, USA

## Abstract

Recent studies have shown that disease-susceptibility variants frequently lie in cell-type-specific enhancer elements. To identify, interpret, and prioritize such risk variants, we must identify the enhancers active in disease-relevant cell types, their upstream transcription factor (TF) binding, and their downstream target genes. To address this need, we built HACER (http://bioinfo.vanderbilt.edu/AE/HACER/), an atlas of Human ACtive Enhancers to interpret Regulatory variants. The HACER atlas catalogues and annotates in-vivo transcribed cell-type-specific enhancers, as well as placing enhancers within transcriptional regulatory networks by integrating ENCODE TF ChIP-Seq and predicted/validated chromatin interaction data. We demonstrate the utility of HACER in (i) offering a mechanistic hypothesis to explain the association of SNP rs614367 with ER-positive breast cancer risk, (ii) exploring tumor-specific enhancers in selective *MYC* dysregulation and (iii) prioritizing/annotating non-coding regulatory regions targeting *CCND1*. HACER provides a valuable resource for studies of GWAS, non-coding variants, and enhancer-mediated regulation.

## INTRODUCTION

Enhancers are distal regulatory DNA regions essential for the precise spatiotemporal control of gene expression (1–6). They are bound by transcription factors (TFs) and activate gene expression through interaction with the gene promoter in a cell-type-specific manner (7). Sequence variants within enhancers can alter transcription factor binding and/or disrupt enhancer-promoter interactions, resulting in gene expression dysregulation and disease (8–11). For instance, three enhancer variants have been shown to reduce *RET* expression by disrupting SOX10, GATA2 and RARB binding and thus increase Hirschsprung disease risk (12). As another example, cancer-risk SNP rs6983267 has been found to increase TCF7L2 binding and enhancer activity to elevate *c-MYC* expression in colorectal cancer cells (13,14). Recent genome-wide association studies (GWAS) have found >88% of disease-risk variants lie in non-coding regions (15), especially enriched in enhancers (16). To identify, interpret, and prioritize enhancer risk variants, we first must identify active enhancers in disease-relevant cell types, their upstream transcription factor binding and their downstream target genes.

**Figure 1.**
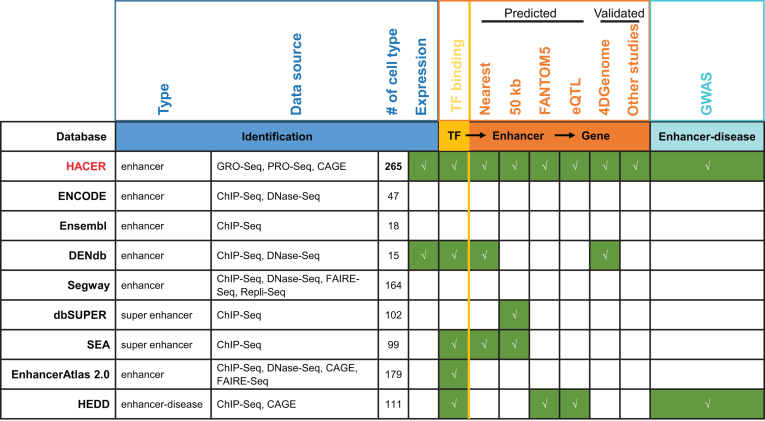
Summary of features distinguishing HACER from exiting enhancer databases.

Genome-wide cell-type-specific enhancers can be identified based on clusters of TF binding and certain histone modification patterns observed in ChIP-Seq and/or based on accessible ‘open’ chromatin identified through DNase-Seq and FAIRE-Seq (17–23). These data types and approaches form the basis of several enhancer databases (24–27) (Figure 1). For example, ENCODE combines DNase and H3K27ac signals to predict enhancer-like regions across 47 human cell types (http://zlab-annotations.umassmed.edu/enhancers/). The Ensembl Regulatory Build applies a genome segmentation algorithm to DNase-Seq and ChIP-Seq datasets for 18 human cell types to assign the regulatory state of each base pair, including enhancers (24). The Segway encyclopedia provides functional elements annotation (such as promoters and enhancers) of 164 human cell types using ChIP-Seq, DNase-Seq, FAIRE-Seq and Repli-Seq (BioRxiv: https:// doi.org/10.1101/086025). DENdb applies five methods to ChIP-Seq histone modification data to predict enhancers in 15 human cell-lines (25). dbSUPER (26) and SEA (27) are two super-enhancer databases that combine ChIP-Seq signals for TF binding and H3K27ac data for 102 and 99 human cell types, respectively.

Recent studies have shown that bi-directional enhancer RNA (eRNA) production, strongly correlated with enhancer activity (28,29), is a more direct and reliable indicator than TF binding or histone markers (30–32). The FANTOM5 project used Cap Analysis of Gene Expression (CAGE) tags to detect 43,011 putative enhancers based on bidirectional eRNA pairs (29). EnhancerAtlas 2.0 (33) and HEDD (34) are two comprehensive enhancer resources, which combine a large number of datasets including ChIP-Seq histone marker data and FANTOM55 CAGE profiles for 179 and 111 cell types, respectively. GeneHancer is a database of enhancer and enhancer–gene associations derived from multiple sources, embedded in the framework of GeneCards (35). Enhancer and enhancer–gene association across cell types are aggregated to generate a confidence score, which makes it difficult to explore cell-type-specific enhancer and interactions.

In comparison to CAGE signals, which are often dominated by highly abundant and stable RNA, nascent RNA sequencing approaches such as GRO-Seq and PRO-Seq are more sensitive to unstable eRNAs, thus offering increased coverage of enhancer regions (36); however, GRO/PRO-Seq data are either not used or not processed in a standard way to identify active enhancers. Even if enhancers are detected, they are scattered in the literature, and have not been collected in any database.

To study enhancer function, one fundamental step is to link enhancers with their upstream regulators and downstream target genes. TF ChIP-Seq provides a map of binding sites in enhancer regions, but connecting enhancers with their target genes remains challenging. The earliest and most common strategy has been to assign enhancers to the nearest gene (28,37–39) or to genes within a certain distance (40,41). Studies have shown, however, that enhancers can skip the nearest gene to regulate a more distal one, and the distance can be quite large (42). Recently, the FANTOM5 project used expression correlation between eRNA and promoters to predict regulatory links (29). The GTEx project employed expression quantitative trait locus (eQTL) analysis to identify the impact on target gene expression of single-nucleotide polymorphisms (SNPs) within an enhancer (43). Compared with predictive approaches, chromosome conformation capture-based technologies (such as 4C, 5C, Hi-C, ChIA-PET, HiChIP and Capture Hi-C) provide more reliable data to find target genes (42). Although rapid progress in these technologies has led to a dramatic increase in chromatin interaction data, existing databases still provide limited information on enhancer-mediated regulation, especially for chromatin contacts detected by high-throughput experiments (Figure 1).

To fill these gaps and facilitate study of regulatory variants, we developed HACER, an atlas of human active and in-vivo-transcribed enhancers. HACER catalogues and annotates 1 676 284 enhancers in 265 human cell lines by integrating FANTOM5 CAGE profiles and reprocessing publicly available GRO/PRO-Seq data. To place enhancers within regulatory networks, HACER identifies 772 902 TF–enhancer bindings based on reanalysis of ENCODE ChIP-Seq data, as well as integrating data for a large number of predicted chromatin interactions, and most importantly, validated interactions from high-throughput experiments. Notably, HACER annotates ∼6.5 million validated enhancer-promoter interactions, ∼1.4 million from 4DGenome and ∼5.1 million from chromosome conformation capture-based technologies (Table 1). HACER provides query tools to interpret disease risk SNPs, to explore disease-specific enhancers and enhancer–gene interactions, and to annotate/prioritize non-coding regulatory variants. HACER further allows for visualization of transcriptional regulatory networks made up of TFs, enhancers, and target genes, along with risk SNPs and eQTL variants. Cell-type-specific knowledge of TF–enhancer–gene interactions bridges the gap between gene regulation and human disease and will greatly facilitate deciphering of the functional role of enhancer variants in disease risk. HACER is available at http://bioinfo.vanderbilt.edu/AE/HACER/.

**Table 1.** Database statistics

HACER	#
Cell line	265
Tissue	42
Enhancer	1 676 284
TF–enhancer binding	772 902
Validated enhancer-promoter interaction	6 460 619
eQTL	1 581 613
GWAS	3 435

## MATERIALS AND METHODS

### Active enhancer identification and quantification

HACER identifies enhancers based on bidirectional eRNA pairs detected in GRO-Seq, PRO-Seq, and CAGE profiles. We conducted an extensive search of GRO/PRO-Seq datasets in the NCBI GEO database and downloaded all available raw sequence data for human cell lines under normal culture conditions. In total, we obtained 211 GRO-Seq and 41 PRO-Seq datasets. After adapter trimming and low quality sequence removal by cutadapt (44), GRO-Seq reads or reverse-complemented PRO-Seq reads from FastX tools (45), longer than 15 bp, were aligned to human genome hg19 using Bowtie2 (46). Reads mapped to rRNA loci and reads with mapping quality less than 10 were removed. NRSA (http://bioinfo.vanderbilt.edu/NRSA/) was applied to identify active enhancers and to quantify their transcription (47). Enhancers detected via CAGE and their associated enhancer expression matrix in each cell type were downloaded from FANTOM5 (29,48). A CAGE enhancer was defined to be active in a cell type if transcribed in that cell type. Enhancer expression was quantified by log_2_-transformed number of reads per bp (log_2_ density). Enhancers annotated in HACER were further cross-referenced to existing enhancer databases, including VISTA (https://enhancer.lbl.gov/) (49), ENCODE Enhancer-like Regions (http://zlab-annotations.umassmed.edu/enhancers), The Ensembl Regulatory Build (https://useast.ensembl.org/info/genome/funcgen/regulatory_build.html) (24), and chromatin state segmentation by ChromHMM from ENCODE/Broad (https://genome.ucsc.edu/cgi-bin/hgTrackUi?g=wgEncodeBroadHmm&db=hg19) (50,51), as well as the super-enhancer database dbSUPER (http://asntech.org/dbsuper/index.php) (26). All the data in HACER is based on genome build hg19.

### TF–enhancer binding

To map TF binding sites within enhancers, we reanalysed ENCODE TF ChIP-Seq data. Seventeen cell lines with active enhancers annotated in HACER have corresponding ChIP-Seq data for 156 TFs in ENCODE. We downloaded peak files from ENCODE and assigned TF peaks in a cell type to active enhancers in the same cell type under the following criteria: the enhancer, instead of a promoter, is closest to the peak; and the enhancer is within ±1 kb from the peak. The TF was then considered to bind the enhancer. Both the closest site to enhancer, and the site with the highest score if multiple peaks were assigned to the enhancer were recorded for each TF–enhancer pair. In total, 772 902 TF–enhancer bindings, involving 156 TFs and 81 478 enhancers, were detected and annotated in HACER.

### Enhancer–gene interactions

HACER provides the most complete resource to-date of enhancer–gene interactions, including 8 525 861 predicted and 6 460 619 experimentally validated links. The predicted interactions are derived from four strategies: (i) assigning enhancers to the nearest gene; (ii) linking enhancers to genes within a distance of 50 kb; (iii) FANTOM5 (29) and (iv) GTEx (V7) (43). FANTOM5 predicts links based on the assumption that transcriptional activity of the enhancer and of the putative target gene transcriptional start site (TSS) are correlated across human cells (29). GTEx uses expression quantitative trait locus (eQTL) analysis to estimate the effect of enhancer variants on gene expression. Experimentally validated interactions were collected from 4DGenome (52) and chromatin interaction studies including Hi-C, ChIA-PET, HiChIP and Capture Hi-C (53–58). 4DGenome is a comprehensive database of chromatin interactions compiled through literature curation, covering both low and high-throughput experimental assays. HACER annotates an additional ∼5.1 million chromatin interactions based on studies not collected in 4DGenome, such as interactions from high resolution Capture Hi-C in two human blood cell types (53).

### Assignment of GWAS SNPs to enhancers

NHGRI-EBI GWAS Catalog data (hg19) was downloaded from https://www.ebi.ac.uk/gwas/downloads (59), and includes SNP ID, disease/trait, *P*-value for the association, odds ratio, and PMID. GWAS SNPs are assigned to HACER enhancers if the SNP falls within an enhancer region.

### Database implementation

The HACER website runs on a Linux-based Apache web server. PHP is used for server-side scripting. The database is organized and managed by MySQL. The web pages are constructed using HTML5 and rendered using the CSS library Bootstrap. A cross-platform JavaScript library jQuery is used to provide a responsive user-friendly front-end interface.

## RESULTS

### Statistics and usage

HACER currently catalogues and annotates 1 676 284 active, *in-vivo*-transcribed enhancers across 265 human cell types. It includes 772 902 TF–enhancer bindings, 6 460 619 experimentally validated enhancer-promoter interactions, 1 581 613 eQTL variants, 66 942 FANTOM5 eRNA-promoter co-expressions, and 3 435 GWAS SNPs (Table 1). A user-friendly web interface was developed to browse, query, and download the enhancer data; to report new GRO/PRO/CAGE datasets; and to send feedback (Supplementary Figure S1).

### Browsing HACER

Users can browse all enhancers by cell line (Supplementary Figure S2A). When the cell line of interest is selected, all enhancers in this cell line are listed in tabular format with the option to filter and customize by annotation (enhancer ID, genomic coordinates, nearest active gene, distance to nearest gene, detection technique, cell type, and/or cross-reference to existing enhancer databases) (Supplementary Figure S2B). Clicking the enhancer ID reveals further detailed information for each enhancer, organized on five tabs for different types of information: basic, TF binding, target genes, GWAS, and eQTLs (Supplementary Figures S2C–G). The ‘Basic’ tab not only provides basic information for the enhancer (name, cell line, location, closest active gene, etc.), but also displays the enhancer sequence, a boxplot illustrating all eRNAs expressed in the cell line with a highlighted dot representing the enhancer RNA level, and cross-reference to existing enhancer databases (Supplementary Figure S2C). The ‘TF binding’ tab lists TF binding sites within the enhancer, including both the closest site and the highest score of the binding site (Supplementary Figure S2D). The ‘Target genes’ tab lists all target genes interacting with the enhancer based on FANTOM5, 4DGenome, or chromatin interaction studies (Supplementary Figure S2E). The ‘GWAS’ (Supplementary Figure S2F) and ‘eQTL’ (Supplementary Figure S2G) tabs present, respectively, GWAS SNPs and eQTL variants falling within the enhancer region.

### Querying HACER

HACER provides four strategies to query the database: SNP-centric, gene-centric, genomic coordinate-centric, and batch query. When queried on a GWAS risk SNP, HACER returns the cell-type-specific enhancers in which the SNP is located, along with their transcription factor binding and target genes. The transcriptional regulatory network of TF–enhancer-target genes is visualized along with the SNP, providing insight into potential mechanism(s) through which the SNP increases disease risk (*Case study 1*). When queried on a gene, HACER returns all enhancers targeting this gene, providing insight into disease-specific enhancers and enhancer–gene interactions (*Case study 2*). When queried on a genomic region, HACER finds active enhancers overlapping the region. Batch query allows the user to input a set of non-coding variants or enhancers, which HACER then prioritizes by their functional importance (*Case study 3*).

#### Case study 1: interpret breast-cancer risk SNP rs614367

Breast cancer is the most common cancer among women worldwide. Genome-wide association studies have successfully identified multiple breast cancer susceptibility loci (60), with rs614367 among those with the strongest association specific to estrogen receptor (ER)-positive disease (61,62). The SNP rs614367 is located in an intergenic region with multiple flanking genes, including *CCND1, MYEOV, ORAOV1, FGF19, FGF4* and *FGF3*, all of which are potential breast cancer susceptibility genes. To find the gene that mediates the association between the SNP and breast cancer risk, we queried HACER on rs614367 and limited the search of active enhancers and chromatin interactions only in MCF7, an ER-positive breast cancer cell line. Within genomic coordinates chr11:69316698–69335855, we found four active enhancers harbouring the SNP. These enhancers are bound by E2F1/GATA3/MYC/TCF7L2/ZNF217 and interact with *CCND1* and *MYEOV* in the MCF7 cell line (Supplementary Figure S3). One eQTL was predicted to affect *GAL* expression; however, this link was detected in colon but not breast tissue (blue line in Supplementary Figure S3). Thus HACER narrowed the target genes to two candidates: *MYEOV* and *CCND1. CCND1* functions as a mediator of estrogen-induced cell proliferation, and *CCND1* expression together with inactivation of pRb are features of tumors with poor response to endocrine therapies (63). Therefore, *CCND1* is highly likely to be the gene that mediates rs614367 breast cancer susceptibility: variants in the enhancer region (either rs614367 or other functional variants) may be hypothesized to drive risk by disrupting E2F1/GATA3/MYC/TCF7L2/ZNF217 binding and affecting *CCND1* expression. This hypothesis has been validated by a recent extensive fine-scale mapping of this region in 89 050 European subjects, which found *EKL4* and *GATA3* as mediators of the regulatory effect of this region on *CCND1* expression (64).

#### Case study 2: explore tumor-specific enhancers targeting MYC

MYC is one of the most commonly activated oncogenes in a broad spectrum of human cancers (65). Dysregulation of MYC is often caused by tumor-specific super-enhancers in the region surrounding the MYC gene (41,66–73). HACER allows for ready exploration of diverse tumor-specific enhancers that regulate the MYC gene. When we query HACER on the MYC gene in any cell type, we find 86 MYC enhancers that are formed within the 3 Mb MYC locus (chr8:127574516–130664889) (Supplementary Figure S4A). If we limit enhancers and enhancer-promoter interactions to HCT-116, a colon cancer cell line, we find seven enhancers upstream of the *MYC* gene (Supplementary Figure S4B); the first four and the last of these previously have been labelled as super-enhancers in HCT-116 (54,69). If we limit the query to K562, a leukemia cell line, we find two enhancers downstream of the MYC gene (Supplementary Figure S4C). One is ∼440 kb downstream of MYC TSS, and the other is located at the distal end of the 3 Mb region (∼1.9 Mb from MYC TSS) and has previously been labelled as a super-enhancer (54). Both regions have been found to regulate MYC expression in mouse leukaemia cell lines (74,75). In short, HACER is able to identify diverse tumor-specific MYC enhancers/super-enhancers, differing in size and location, and consistent with previous studies (54,76).

#### Case study 3: prioritizing non-coding regulatory variants

Identification of non-coding drivers from thousands of variants is difficult. HACER prioritizes non-coding regulatory variants based on two assumptions: (i) variants within enhancer elements targeting disease genes are highly likely to be drivers, and (ii) regions with more annotation available are more likely to be functional. When queried on a list of non-coding regions (batch query) and a set of disease genes, HACER highlights the regions targeting the disease genes and ranks all regions by the availability of information on cell-type-specific enhancers, TF binding, target genes, and GWAS risk SNPs. As an example shown in Supplementary Figure S5, HACER is queried on a list of non-coding regions, including a breast cancer risk region (chr11:69328760–69328765), and ‘CCND1’ as the target gene of interest. HACER highlights the breast cancer risk region targeting *CCND1* and ranks all query regions by the availability of functional information (Supplementary Figure S5B). A network view is also provided to show upstream TFs and downstream targets of each query (Supplementary Figure S5B).

### Downloading enhancers, reporting new datasets and sending feedback

HACER provides a Download page for users to download all enhancers by cell type. To download enhancers, click on the cell type of interest; the resulting file lists every enhancer in this cell type with comprehensive genomic annotation (e.g., chromatin location, target genes, detection methods). HACER also provides a Contact page for users to report new PRO-Seq, GRO-Seq and CAGE datasets. A hyperlink to the raw data should be provided, from which we will download, and process the data to identify, annotate and deposit enhancers into HACER. Users also are encouraged to send feedback via the Contact page.

## DISCUSSION AND FUTURE DEVELOPMENT

HACER not only catalogues and annotates active, *in-vivo*-transcribed enhancers from a large number of human cell types, but also provides insight into enhancer-mediated regulation. Integrating active enhancer, TF–enhancer binding, and enhancer–promoter interaction data within a single data repository enables the study of complex transcriptional regulation that drives cell-specific gene activities. HACER provides tools to find the potential mechanism underlying the association of enhancer variants with disease risk, to study enhancer activity and enhancer–gene regulation across different cell lines, and to link non-coding variants with disease genes for narrowing down potential causal variants. HACER represents a valuable and unique resource for studies on regulatory variants and enhancer-mediated regulation.

Few cell lines have matched GRO/PRO/CAGE, TF ChIP-Seq and chromatin interaction data, which greatly limits the ability to study enhancer function in a cell-type-specific manner and to explore enhancer roles in human disease. We will update HACER immediately if new datasets are reported through the contact page. Meanwhile, we plan to update HACER semi-annually to match the latest GRO/PRO/CAGE-Seq and chromatin interaction data from public databases and literature. Because enhancers and TSSs both produce bidirectional transcripts, enhancers (especially long enhancers) can be difficult to distinguish from alternative TSSs or long non-coding RNAs (lncRNAs) (36), resulting in possible false positive enhancer calls. In the future, we plan to add histone marker H3K4me3 and H3K4me1 along with RNA-Seq data to help distinguish enhancers from novel TSSs and to reduce false positives.

Disruption of enhancer function leads to disease via alteration in target gene regulation. Currently, HACER links enhancers with disease based on disease-associated genetic variants from the GWAS Catalog; gene-disease association data have not been integrated. To study the role of enhancers in human disease, we plan to add gene-disease association data from MalaCards (77), DISEASES (78) and DisGeNET (79) and provide tools to explore enhancer-disease association directly.

## Supplementary Material

Supplementary Data
