# Supplementary material for: HACER: an atlas of human active enhancers to interpret regulatory variants
Source: Nucleic Acids Res. 2018 Sep 24;47(Database issue):D106–12. doi: 10.1093/nar/gky864 (PMC6323890; doi:10.1093/nar/gky864)
Supplement: Supplementary Data [file gky864_supplemental_files.pdf]

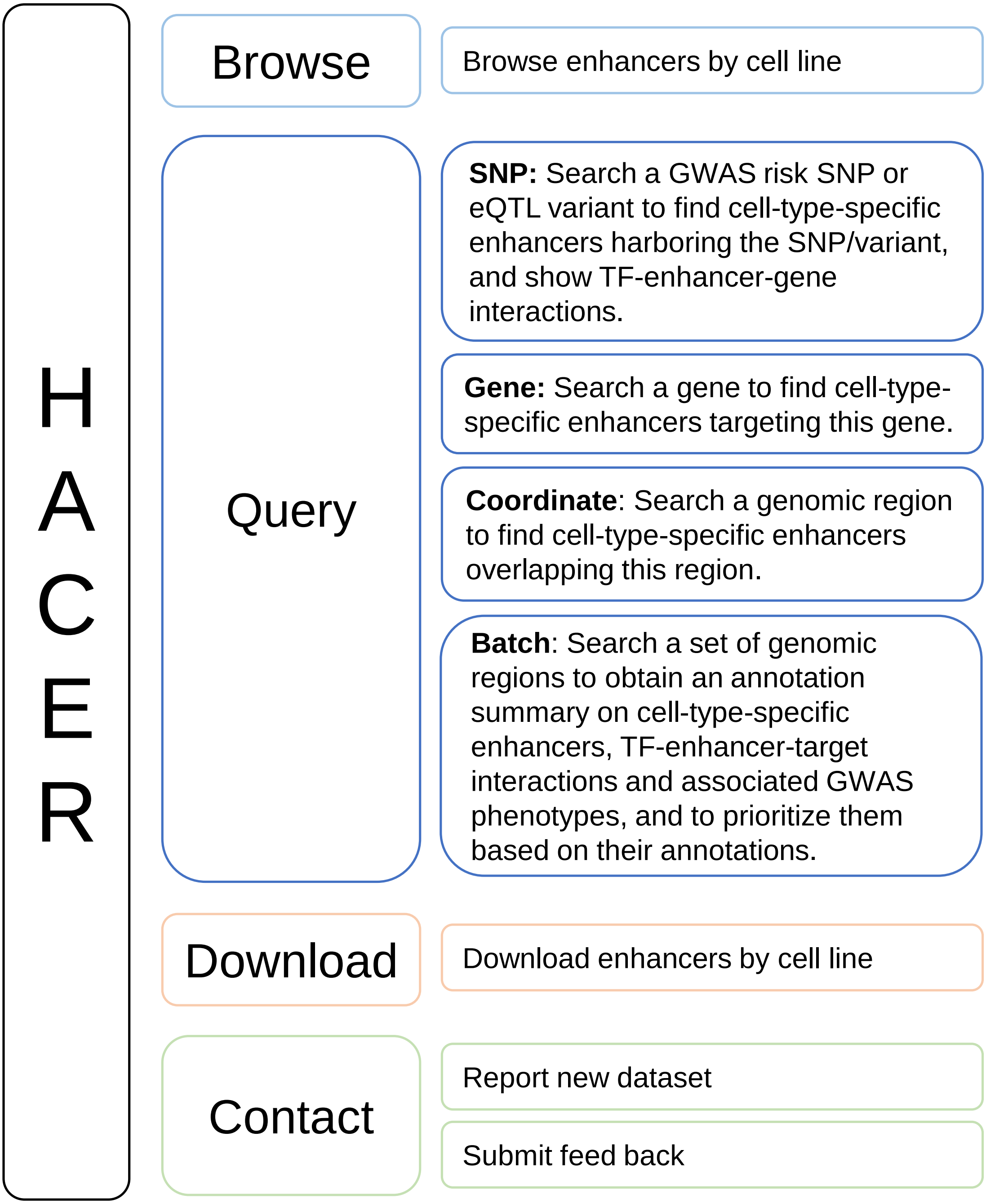

Supplementary Figure S1. Functionality of HACER. Functionality of HACER includes browse, query, download and contact. The query function provides four ways: SNP-centric, gene-centric, coordinate-centric, and batch query.

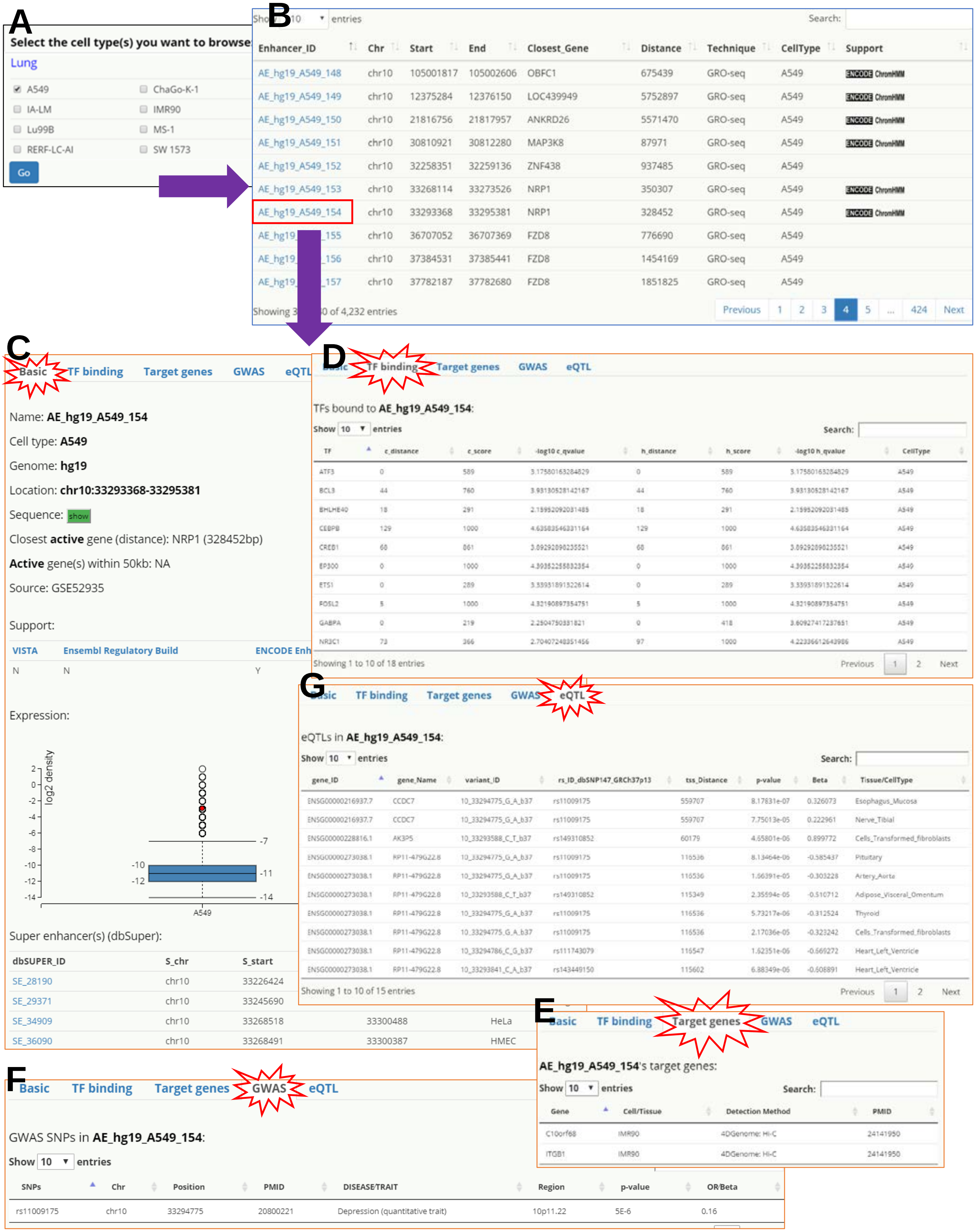

Supplementary Figure S2. Screenshot of browsing HACER. (A) Select the cell type for browsing. (B) List of enhancers in the cell type. (C-G) Detailed information of a selected enhancer, including basic information (C), TF binding (D), target genes (E), NHGRI-EBI GWAS SNP hits (F), and GTEx eQTL traits (G).

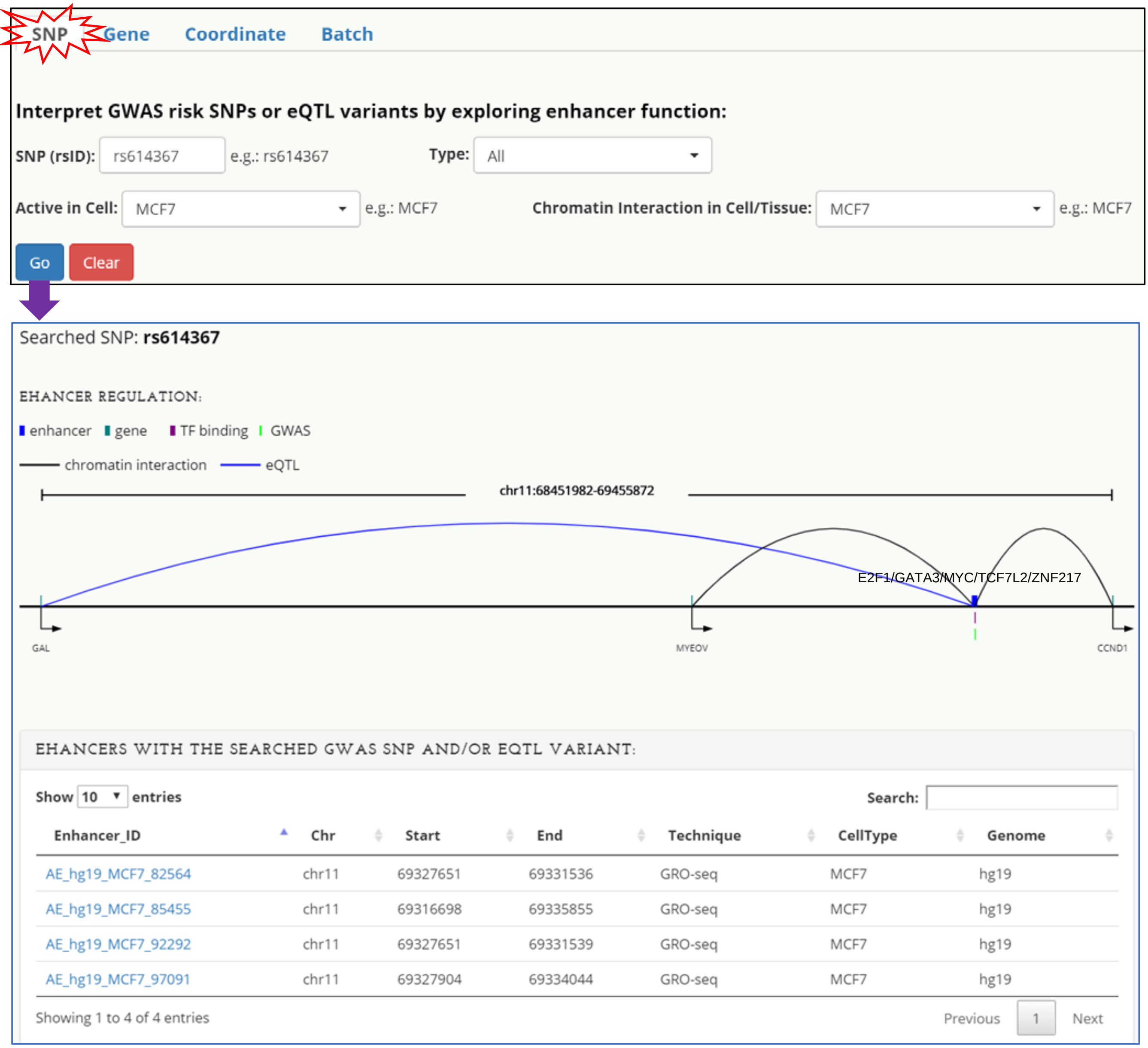

Enhancer_ID	Chr	Start	End	Technique	CellType	Genome
AE_hg19_MCF7_82564	chr11	69327651	69331536	GRO-seq	MCF7	hg19
AE_hg19_MCF7_85455	chr11	69316698	69335855	GRO-seq	MCF7	hg19
AE_hg19_MCF7_92292	chr11	69327651	69331539	GRO-seq	MCF7	hg19
AE_hg19_MCF7_97091	chr11	69327904	69334044	GRO-seq	MCF7	hg19

Supplementary Figure S3. An example of interpreting the association of SNP rs614367 with breast cancer risk. (A) Query HACER by SNP rs614367 in MCF7 cell line. (B) Enhancer-mediated regulatory network, which shows enhancers (blue) harbouring the SNP (lime), their upstream TF binding (purple) and downstream targeted genes (teal). Enhancer-gene interactions are denoted by black lines if experimentally validated or blue lines if predicted from eQTL analysis.

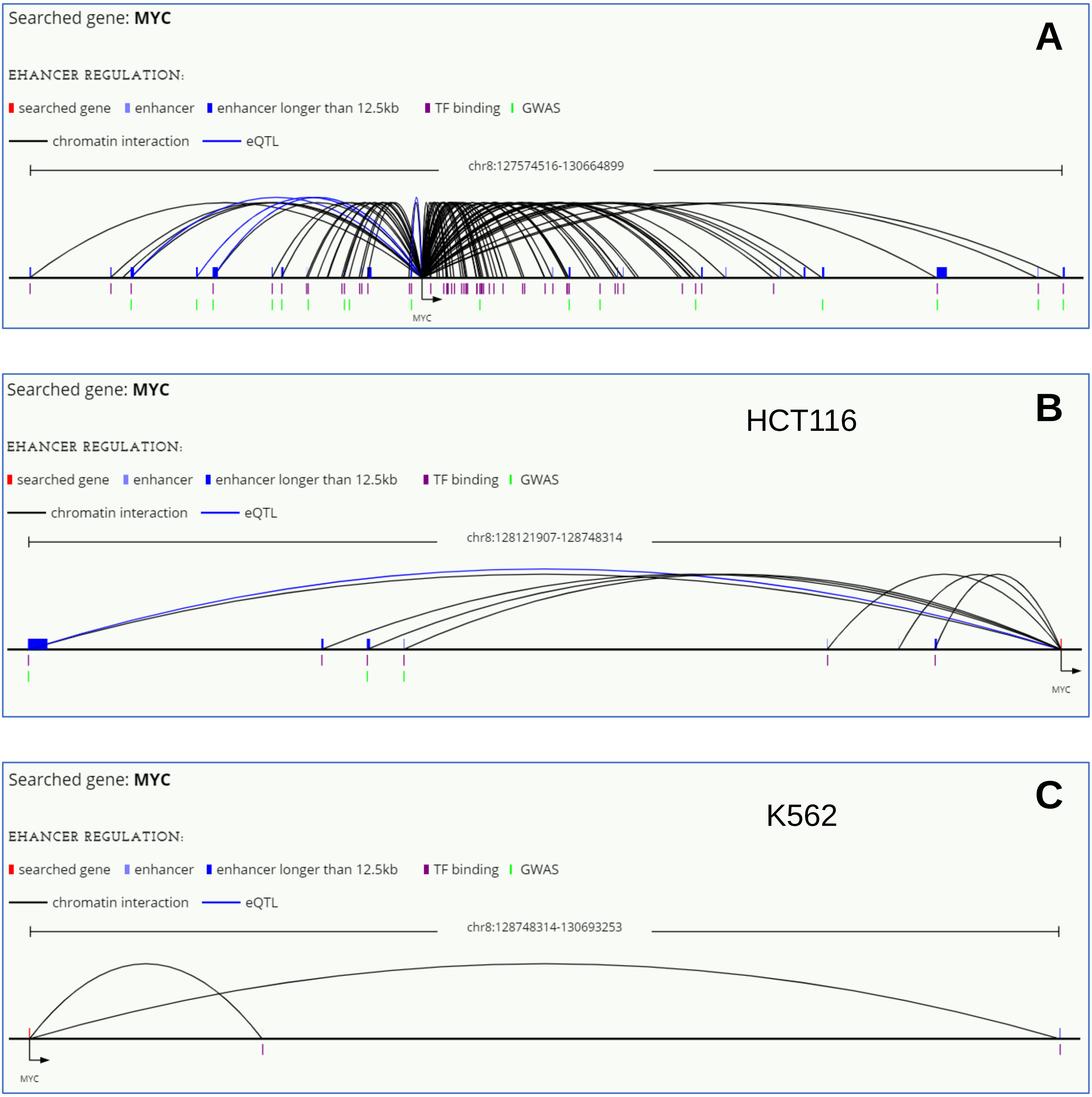

Supplementary Figure S4. An example of exploring tumor-specific enhancers targeting MYC. Query HACER by MYC gene in all cells (A), only in HCT116 cell (B) or in K562 cell (C). Enhancers (light blue or blue boxes) targeting MYC, along with their upstream TF binding (purple) and GWAS risk SNP (lime) are shown. Enhancer-MYC interactions are denoted by black lines if experimentally validated or blue lines if predicted from eQTL analysis.

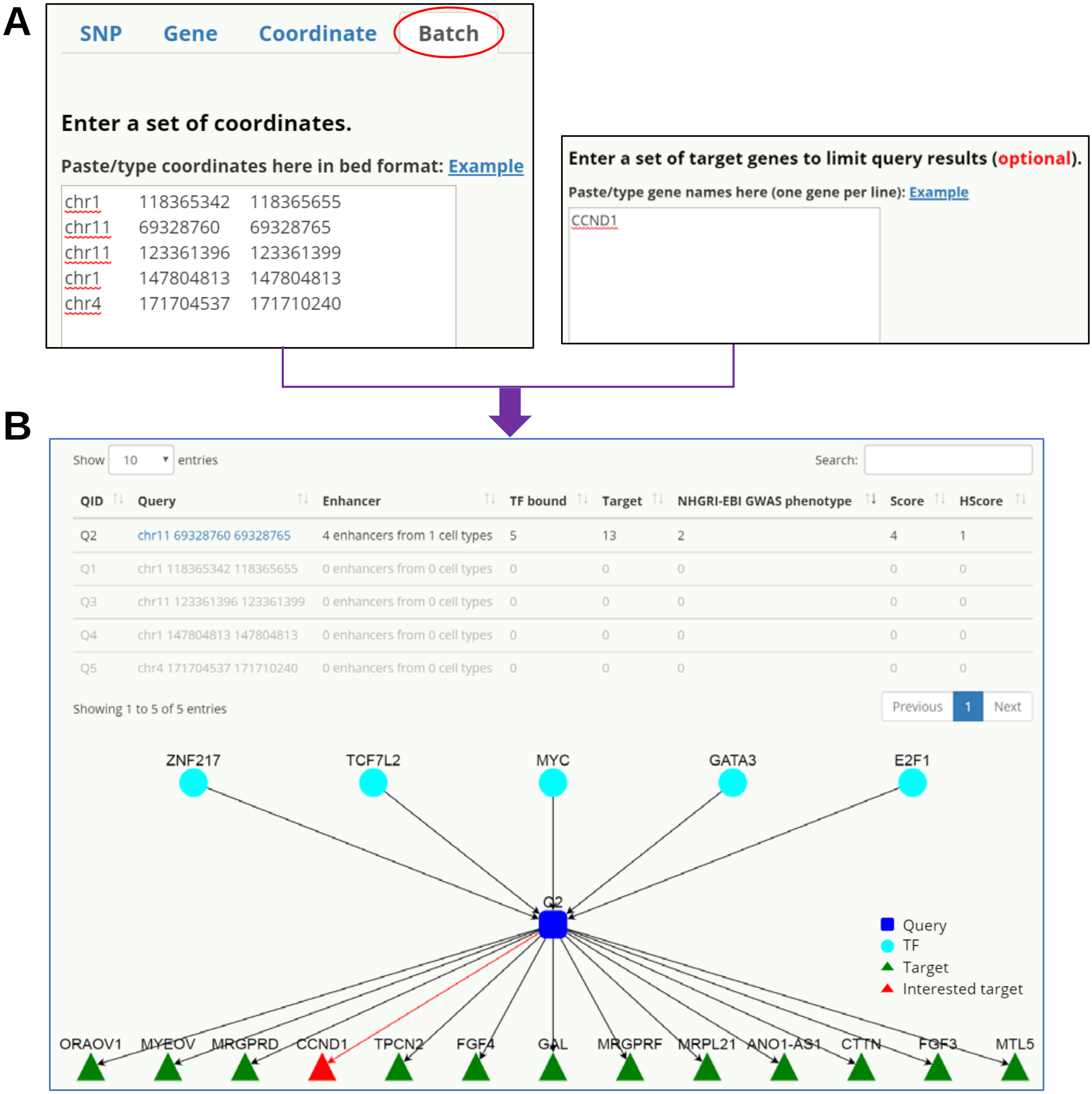

Supplementary Figure S5. An example for prioritizing/annotating non-coding variants/enhancers. (A) Query HACER by a set of coordinates (locations of enhancer or non-coding variants) and a set of target genes. (B) Queries can be ranked by the number of available functional evidence (Score) or by the number of target genes of interest (HScore) (top panel). A network is provided to show upstream TFs (cyan circle) and downstream targets (green triangle) of queries (blue rectangle) with interactions and genes of interest highlighted in red.(bottom panel).
